# A Scalable Workflow for Extracting Species Interactions From Ecological Literature Using Large Language Models

**DOI:** 10.1002/ece3.74626

**Published:** 2026-10-27

**Authors:** François Keck, Henry Broadbent, Florian Altermatt

**Affiliations:** ^1^ Department of Evolutionary Biology and Environmental Studies University of Zurich Zürich Switzerland; ^2^ Department of Aquatic Ecology Eawag, Swiss Federal Institute of Aquatic Science and Technology Dübendorf Switzerland

**Keywords:** biological interactions, large language models, prompt engineering, relation extraction, structured data extraction, taxonomic entity linking, taxonomic named entity recognition, text mining

## Abstract

The contemporary ecological crisis calls for integration and synthesis of vast amounts of mostly scattered and often hard‐to‐extract ecological information that is dispersed across hundreds of thousands of scientific papers. Recent advancements in natural language processing (NLP) and the emergence of large language models (LLMs) offer a novel and promising approach to this persistent challenge. Here, we present a replicable and adaptable workflow that automates the extraction of a broad range of biological interactions among species from scientific literature using LLMs. We provide a demonstration of this workflow by performing a large‐scale data extraction directly from a corpus of 83,910 scientific articles using OpenAI GPT‐4o. Our approach successfully extracted a network of 144,402 interactions between 36,471 taxa. Performance analysis shows that the model exhibits a precision of 89.5% with a recall of 70.0%, demonstrating that a general‐purpose LLM can perform complex ecological information extraction at scale for analysis and synthesis. Results depend on quality filtering choices and inherit biases present in the underlying literature, emphasizing the need for validation and quality control. Standardized outputs can be browsed, queried, or joined with existing interaction repositories and taxonomic backbones, facilitating downstream reuse and interoperability. Our case study shows that LLMs are capable of carrying out complex extraction tasks on key ecological data on a very large scale, paving the way for a multitude of potential applications in ecology and beyond.

## Graphical abstract

We present a replicable workflow that uses large language models to extract biological interactions among species from ecological literature automatically. Applied to a large corpus of scientific articles, the workflow extracted 144,402 interactions among 36,471 taxa. These results show that general‐purpose LLMs can support large‐scale ecological data synthesis by transforming scattered information from the literature into reusable and interoperable datasets.

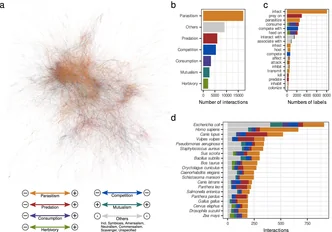

## 1 Introduction

“In nature, nothing exists alone.” This statement by Rachel Carson (1962) very accurately captures that ecological systems are not defined by the sheer number of species, but rather their interactions and dependencies. The field of ecology is fundamentally the study of interactions among organisms and between organisms and their environment. These biological interactions, such as predation, competition, parasitism or mutualism, form the foundation of population dynamics, community composition, and ecosystem functioning (Elton 1927; Paine 1966; Seibold et al. 2018). Biological interactions, together with species distribution and functional traits, also represent the key critical information for understanding and responding to increasing environmental challenges (Pollock et al. 2020), including climate change (Hegland et al. 2009), biodiversity loss (McDonald‐Madden et al. 2016; Sandor et al. 2022), and emerging pathogens (Wardeh et al. 2021).

Yet, data on biological interactions are among the hardest to get hold of (Hortal et al. 2015; Poisot et al. 2021). Our knowledge of the different ways in which species interact is largely based on original natural history observations scattered across a myriad of publications and often dispersed across different research fields (Farrell et al. 2024). Also, the possible number of interactions is vastly larger than the sum of species involved. Imagine a community of 200 plants and 600 insect herbivores–realistic numbers for even a mid‐diversity ecosystem–creating the potential of 19,900 pairwise competitive interactions between the plants and close to 120,000 herbivorous interactions alone. Despite only a very sparse part of these potential interactions being realized, assembling and integrating data on species interactions has been a major challenge, with only a few food webs being largely resolved (Pearse and Altermatt 2013a, 2013b), let alone moving beyond pairwise interactions (Levine et al. 2017).

Because of the central importance of biological interactions for modern ecology and for biodiversity prediction in the current context of global change (Burkle and Alarcón 2011; Cámara‐Leret et al. 2019; Levine et al. 2017; Staniczenko et al. 2017; Wisz et al. 2013), large database projects have been developed with the aim of collecting machine‐readable species‐interaction data (Maiorano et al. 2020; Poelen et al. 2014; Poisot et al. 2016). However, these databases ultimately rely on manual or semi‐automatic extraction and integration of data shared by scientists. While data on species occurrences are rapidly increasing and are expected to become less of a limiting factor for ecological research (Feng et al. 2022), information on species interactions remains significantly lacking and incomplete (Cameron et al. 2019; Hortal et al. 2015; Poisot et al. 2021). This discrepancy arises partly due to the inherent complexity of sampling species interactions (Chacoff et al. 2012) and partly because such data are less systematically recorded and reported in structured databases.

Integrating and synthesizing ecological knowledge is notoriously difficult. This is largely due to how ecology and natural history findings are disseminated. Typically, they are communicated through scientific articles, which often consist of large volumes of unstructured text (Nunez‐Mir et al. 2016). Consequently, the sum of ecological knowledge generated over more than a century, and which we need to mobilize to tackle the global ecological crisis, is dissolved in a vast ocean of text. Unlike structured databases or standardized formats, scientific articles vary widely in their organization. This has made extraction of information laborious and time‐consuming or even impossible, such that most information is still hidden (if not lost) in text. Also, ecological research spans diverse subfields and methodologies, resulting in a large and heterogeneous body of literature that can be difficult to navigate and integrate. Inconsistent terminology and concepts, and lack of general schemas for databases across authors and subfields also hinder synthesis (Hodges 2008; Trombley and Cottenie 2019). As a result, ecologists face the daunting task of sifting through a myriad of articles, extracting relevant data, and synthesizing findings into cohesive reviews, databases, and meta‐analyses. Cataloging interactions among species started with the very first steps of ecology by Alexander von Humboldt (Humboldt and Bonpland 1805), yet despite generations of ecologists and naturalists collecting and documenting these interactions, they have proven difficult to integrate in a consistent and inclusive manner.

Automated text extraction has a substantial history in ecology and biodiversity science, using a variety of approaches (Abdelmageed et al. 2022; Farrell et al. 2024; Thessen and Parr 2014) and including applications to literature mapping, food‐web construction and automated content analysis (e.g., Millard et al. 2020; Nunez‐Mir et al. 2016; Tamaddoni‐Nezhad et al. 2013). These works demonstrated the value of text mining for synthesizing ecological knowledge but tools often required substantial task‐specific development and remained limited in their capacity to process heterogeneous and inconsistently structured research outputs (Cornford et al. 2022). Recent advances in deep learning and general‐purpose large language models (LLMs) therefore represent a natural extension of this trajectory, potentially increasing the scale and flexibility of information extraction across diverse scientific corpora (D'Souza et al. 2025; Farrell et al. 2024; Rafiq et al. 2025; Volkanovska 2025). These models trained on vast amounts of text data (Kaplan et al. 2020), now exhibit unprecedented capabilities in understanding and generating human‐like language (Ouyang et al. 2022; Wei et al. 2022), allowing researchers to tackle more complex extraction tasks which until now were limited to highly structured and codified textual data (e.g., collections of taxonomic descriptions; Coleman et al. 2023).

Early academic discussion of general‐purpose LLMs, including ChatGPT, frequently focused on text generation, such as publication writing (Hutson 2022) and education (Campbell et al. 2024; Cooper et al. 2024; Kasneci et al. 2023). However, LLM‐based retrieval and structured information extraction now constitute active areas of methodological development and application across scientific domains. Notable case studies in ecology have applied LLMs to plant pathogen reports (Gougherty and Clipp 2024), research abstracts (Castro et al. 2024; Scheepens et al. 2024), news reports (Castro et al. 2024), conservation evidence databases (Iyer et al. 2025), citizen science annotations (Zou et al. 2026) and bee–pesticide studies (Parkinson et al. 2026). The generative capacity of LLMs has also been used to automate species grouping and food web parameterization (Spillias et al. 2026) and to build chatbot applications to interact with scientific data (Gallois et al. 2025).

Here, we demonstrate the capacity of LLMs to extract biotic interactions between species directly from the published literature at a very large scale. Biological interactions are a critically important piece of information for ecological research, yet interaction data remain sparse and unevenly documented. This scarcity is a recognized bottleneck for building and analyzing interaction networks and for improving biodiversity predictions under global change (Pollock et al. 2020), despite ongoing database efforts that still rely heavily on manual extraction (Poelen et al. 2014; Poisot et al. 2021).

Additionally, biological interactions are also an interesting case study, as they represent a technical challenge for automatic data extraction methods. An interaction is complex information involving two actors (two species) and a directional relationship (the interaction). The algorithm or model has to isolate and qualify these elements, despite the extensive vocabulary and sometimes ambiguous terminology (Hodges 2008; Trombley and Cottenie 2019) that covers the wide range of biological interactions and the organisms involved. While dictionary‐based approaches have been developed to detect biological interactions (Muñoz et al. 2019), their efficiency is strongly limited when confronted with the complexity of the language and the multiplicity of syntactic constructions used in the literature. To date, this complexity precluded any attempt at automated extraction of biological interactions on a large scale. Our primary goal is thus a methodological and conceptual proof‐of‐principle, to validate that LLMs can effectively address these extraction challenges. This will enable and foster future large‐scale ecological syntheses and analyses.

## 2 Material and Methods

### 2.1 A Workflow for LLM‐Assisted Biological Interaction Extraction

We adapt a multi‐stage workflow template to convert a large literature corpus into structured, analysis‐ready ecological records (Figure 1). First, corpus filtering and preprocessing select relevant text units and normalize them to reduce processing costs and limit false positives. Second, an LLM extracts relevant information from these text units into a predefined structured schema using an optimized prompt template. Third, postprocessing and harmonization apply automated quality‐control checks and standardization to filter spurious outputs and merge duplicates. Finally, fourth, the performance of the workflow and the quality of the extracted data are estimated by manually reviewing the outputs for a sample of the submitted data.

**FIGURE 1:**
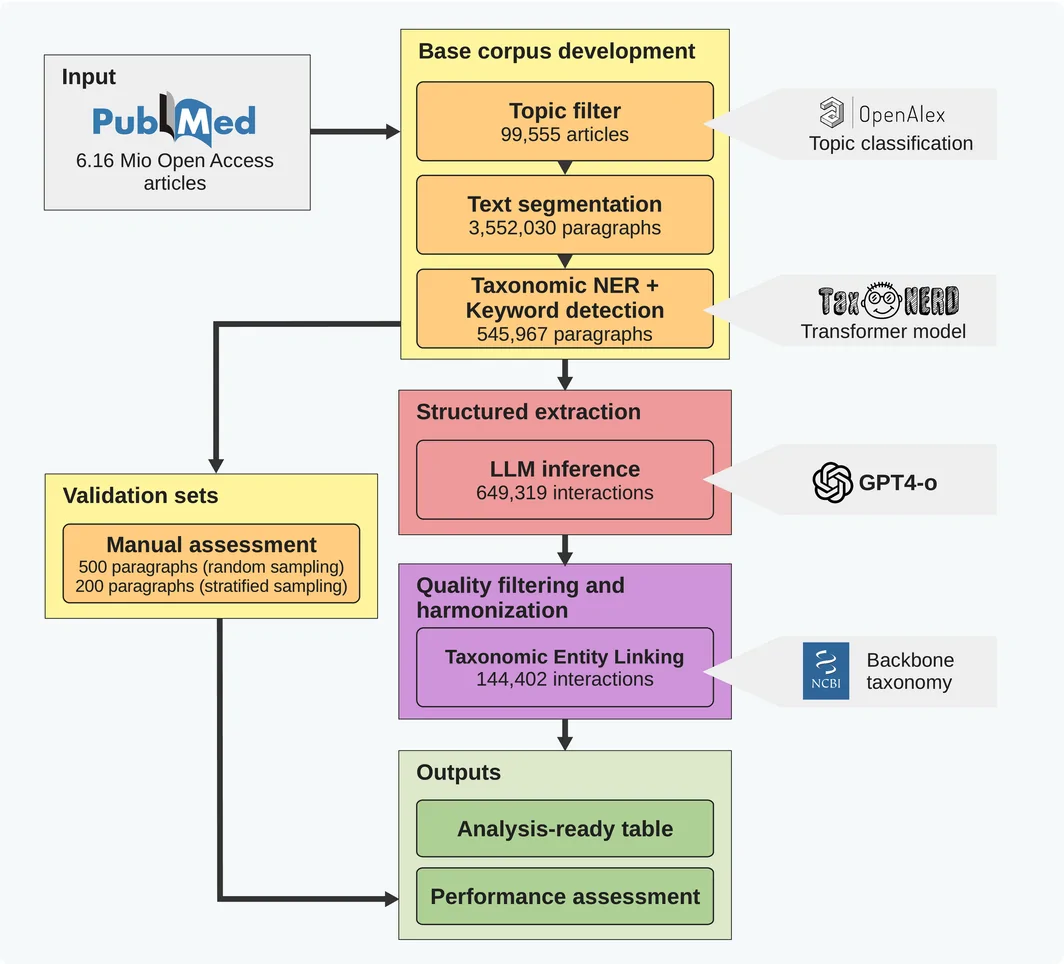
Diagram describing the workflow for LLM‐assisted extraction of biological interactions. For each step, the number and type of records is shown, along with the external tools and data involved.

Below we describe an end‐to‐end implementation of these building blocks for biotic interaction extraction at scale. All data extracted and all code used for analysis are openly available on GitHub (https://github.com/fkeck/gpt_interactions) and archived on Zenodo (https://doi.org/10.5281/zenodo.21397883).

### 2.2 Corpus Filtering and Preprocessing

The text corpus was sourced from the PubMed Central (PMC) Open Access database, which contains millions of publications spanning various scientific disciplines distributed under varying Creative Commons or comparable reuse terms permitting data mining (Roberts 2001). We note that, at the time of processing, OpenAI API inputs were not used for model training by default. The extracted interaction records represent factual assertions rather than reproductions of the source articles' expressive content. To narrow the scope of our analysis, we first refined our corpus to focus specifically on publications related to ecological sciences. Using the OpenAlex database (Priem et al. 2022) and its topic classification system, we identified 99,555 publications tagged under the subfield “Ecology” from an initial pool of 6,159,719 PMC publications. We downloaded the corresponding 99,555 XML files from PMC and parsed them to extract 3,552,030 paragraphs for further analysis. We used paragraphs as the extraction unit because they generally retain enough local context to resolve taxonomic mentions and interactions expressed across multiple sentences. Compared with sentences, paragraphs reduce the risk of contextual loss. In contrast, processing full articles would introduce substantial irrelevant context and many competing taxonomic entities, reducing the signal‐to‐noise ratio and significantly increasing the token consumption.

We refined this dataset by retaining only the paragraphs that included at least two distinct taxonomic names and one keyword associated with species interactions. To identify taxonomic names, we employed TaxoNERD (Le Guillarme and Thuiller 2022), a named‐entity recognition (NER) tool able to recognize both scientific and vernacular taxonomic names. TaxoNERD uses state‐of‐the‐art deep neural models (transformer architecture) and significantly outperforms other approaches on ecological test corpora, reaching F1‐score > 90%. TaxoNERD detected a total of 8,160,227 taxonomic names and at least one taxonomic mention in 1,925,414 paragraphs spanning 97,681 articles. In parallel, we performed a regular expression‐based search to detect species interaction terms using a curated list of 43 relevant keywords and their variations (Data S1). This list was built by adapting the controlled vocabulary used in the mangal (Poisot et al. 2016) and GLOBI (Poelen et al. 2014) databases. The application of both filters—requiring a minimum of two distinct taxonomic names and one species interaction keyword—resulted in a final corpus consisting of 545,967 paragraphs from 83,910 articles.

### 2.3 Prompt Design and Data Extraction

The final dataset of 545,967 paragraphs was subsequently processed using the OpenAI GPT‐4o (v. 2024‐05‐13) model. GPT‐4o is a transformer‐based LLM developed by OpenAI and is accessed via prompt‐based interaction to analyze and generate text in response to natural language instructions (OpenAI et al. 2023). Each paragraph was incorporated into a standardized prompt template (Figure 2), which was then submitted to the OpenAI API for analysis. The temperature of the model was set to zero. All other parameters were set to the default API values (see Methods S1). All the queries were submitted to the server in batches of 50,000. None of the queries resulted in an error. The prompt template was specifically designed to extract all pairwise species interactions present within the paragraph and structure the output in a tabular format. The prompt was developed through an iterative, qualitative process rather than by means of systematic variant testing. During development, model outputs were continuously assessed to identify strategies that consistently improved response quality (i.e., completeness of interaction extraction, appropriate interaction‐category classification, adherence to output schema). Based on these assessments, the final prompt was constructed to incorporate several effective techniques. First, a chain of thought and multistep prompting structure was adopted to support complex reasoning (Wei et al. 2023). Second, one‐shot learning using a clear and representative example was added to guide and optimize the model behavior (Brown et al. 2020). Third, we made an extensive use of assertive prompting by using a mixture of imperative phrasing, uppercase emphasis and exclamation points to highlight important points, in particular those less captured by the model (Amatriain 2024). Fourth, the complex data structure of the output was described through an explicit schema using a widely adopted programming language (Typescript), enabling a more accurate parsing by the model (Yurtsev 2023/2024). Finally, to mitigate reward hacking and promote concise outputs, verbosity was explicitly discouraged in the prompt formulation (Nayab et al. 2025).

**FIGURE 2:**
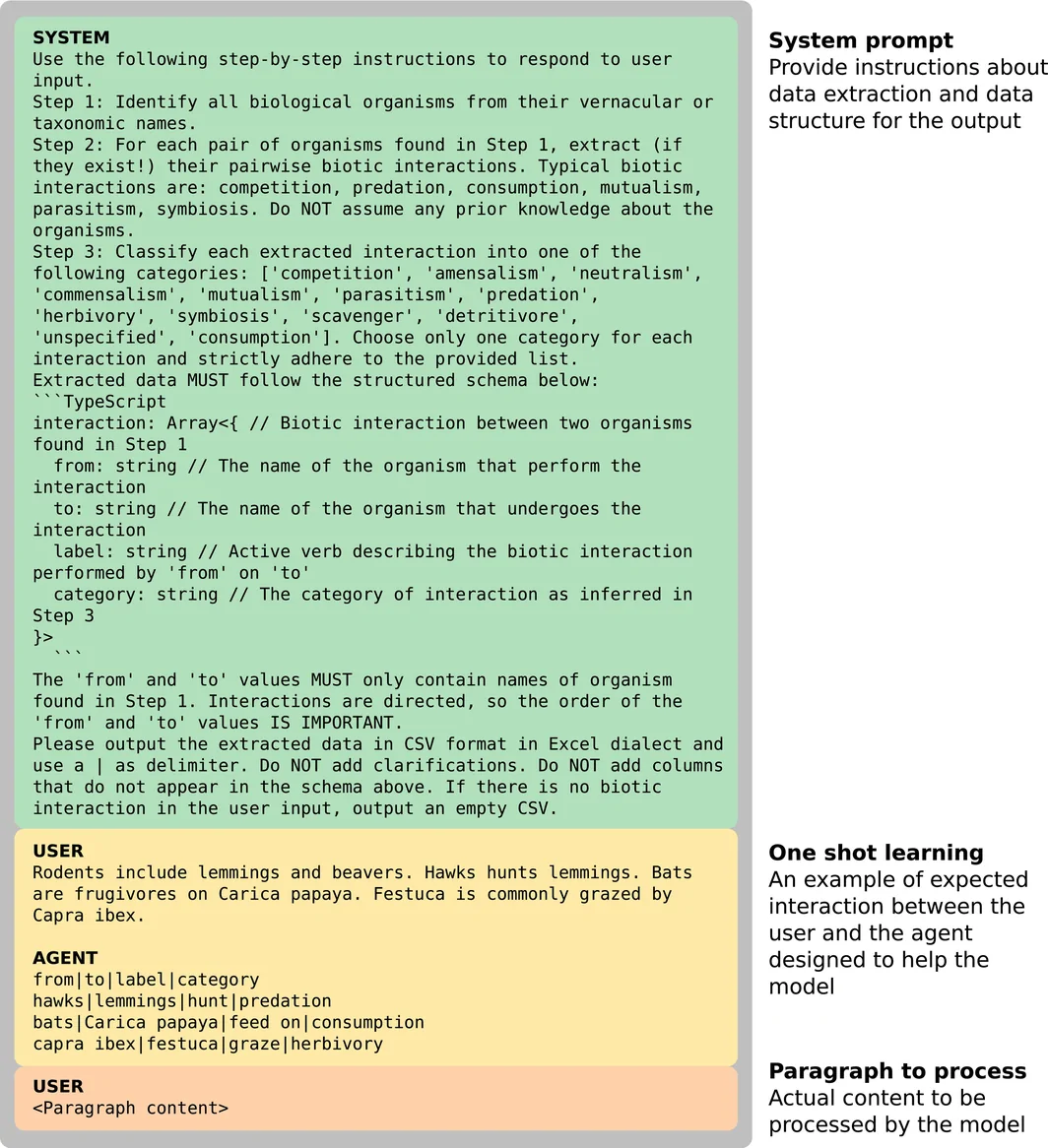
Standardized prompt template used to process each paragraph. First, a system prompt is used to give a detailed description of the task to the model. Second, one example of task resolution is provided to the model to refine performances. Finally, the content of the actual targeted paragraph is provided for extraction.

The output consisted of a four‐column table where the first two columns contained the names of the two species involved in the interaction, the third column specified the nature of the interaction as labeled in the text, and the fourth column classified the interaction into its respective category from the list used in the mangal database (Poisot et al. 2016). This structured format allowed the systematic analysis and categorization of species interactions within the corpus.

### 2.4 Postprocessing and Filtering via Taxonomic Entity Linking

To identify interactions involving entities that cannot be linked to living organisms and to harmonize organism names and link them to their upstream taxonomy, we performed taxonomic entity linking (TEL). TEL consists in mapping extracted named entities to corresponding unique identifiers in a target knowledge base, in our case the NCBI Taxonomy (Federhen 2012). For example, through TEL, the entities dog, domestic dog, *Canis familiaris* and *Canis lupus familiaris* are all linked to the same NCBI Taxonomy ID: 9615 and constitute a unique node in the final network. We implemented TEL through a custom multistep algorithm using data from NCBI, the Encyclopedia of Life (EoL), the Integrated Taxonomic Information System (ITIS), and Wikipedia (see corresponding R script for detailed implementation). The performance of our TEL algorithm was assessed using a random sample of 1000 named entities from our dataset and their corresponding NCBI link, when the latter could be found.

### 2.5 Performance Assessment

To estimate the quality and completeness of the information extracted by our approach, we compared paragraph‐level ground‐truth (annotated directly from the text) against the final, post‐TEL interaction records produced by the workflow, thus the reported performances reflect end‐to‐end performance including TEL‐based filtering. First, we manually reviewed and annotated 500 paragraphs (≈1% of the total corpus) taken at random from all those submitted to the model. Second, to better capture performance across both common and rare taxa, we additionally evaluated a stratified set of 200 paragraphs constructed to achieve balanced representation across taxonomic groups. Strata membership was assigned using the workflow's outputs and ensured that no taxon appeared more than once in this set, whereas ground‐truth interaction presence, species pairs, labels, and categories were manually annotated. Because this stratified set is conditional on successful automated detection, its metrics should be interpreted as performance given detection (a taxonomic‐coverage stress test) rather than an unbiased estimate of global recall. All annotations were performed by a single annotator with a background in ecology, which supported the interpretation of interactions described using discipline‐specific terminology. Because the validation sets were not independently annotated by multiple annotators, annotation variability could not be directly quantified. The annotation process took around 6 days of work for each subset. Extracted and manually annotated records were matched at the paragraph level. Taxonomic synonyms were treated as equivalent when they could be resolved to the same NCBI Taxonomy identifier. Interaction direction was required to match the direction expressed in the source text, and duplicate records representing the same taxon pair and interaction category within a paragraph were counted only once. Exact agreement in taxonomic rank was not strictly required: broader taxa were accepted when they remained compatible with the organism named in the source text. Negated interactions were not annotated as present. Speculative or ambiguous statements were assessed case by case and included only when the paragraph presented the interaction as occurring rather than merely hypothetical. Using these human annotated data, we computed three performance statistics. First, the precision, calculated as the fraction of relevant retrieved interactions among all the retrieved interactions; second, the recall, calculated as the fraction of relevant interactions that were retrieved; and third, the F1‐score (the harmonic mean of precision and recall). These three statistics are those typically used to evaluate information extraction tasks (Resnik and Lin 2010). We also report component‐wise performance for the three core components making up a complete interaction annotation, namely the species pair recognition, the interaction label, and the interaction classification. Component‐wise precision, recall, and F1 scores were computed as interaction‐instance (micro) scores. For each extracted record, a component (species, label, and category) is counted as a true positive if it matches the manual annotation and false positive otherwise, while manually annotated interactions with no corresponding extracted record are counted as false negative. Confidence intervals for all performance estimates were obtained via grouped bootstrap resampling (1000 samples) using source paragraphs as groups.

Finally, because LLM outputs are non‐deterministic, we quantified the repeatability and stability of the results generated by the workflow by re‐running the extraction on a randomly sampled subset of 100 paragraphs 100 times each using identical prompt and API settings. For each paragraph and each extracted interaction, we computed the proportion *p* of replicate runs in which the interaction was produced, and summarized agreement as the expected probability that two randomly drawn runs agree on presence/absence: *A* = *p* ^2^ + (1 − *p*)^2^.

## 3 Results

In total, 545,967 paragraphs originating from 83,910 scientific articles sourced from the PMC Open Access database (Roberts 2001) were processed using the OpenAI API. The extraction runs used 414,029,114 prompt tokens and 12,812,317 completion tokens, which corresponds to a total cost of ~USD 581.6 (OpenAI batch‐mode pricing as of February 2026). The GPT‐4o model combined with our optimized prompt (Figure 2) extracted 649,319 potential biotic interactions. Each of these interactions contained the names of the two entities (theoretically—and ideally—representing names of organisms) involved in the interaction, the nature of the interaction as labeled in the text, and a broad classification of the interaction (e.g., predation, competition, parasitism) performed by the model. In total, all the extracted interactions involved 157,354 unique named entities among which 70,841 could be taxonomically linked to the NCBI Taxonomy database through TEL (Rao et al. 2013).

Across the subset used for TEL benchmarking (1000 named entities), our algorithm linked 593 names to a NCBI taxonomy ID, of which 98.31% were found to be correct. Further, we could confirm the initial observation that many extracted names could not be linked to a specific taxon. Overall, we found that the majority of names that could not be linked did not actually refer to organisms (but for example to gene names). Further causes of links not being established were ambiguities in the naming, misspellings, and gaps in the databases used (Table 1).

**TABLE 1:** List of possible reasons for a non‐match of LLM extracted names in the NCBI taxonomic database (taxonomic entity linking, TEL).

Reasons for TEL non‐match	Example	Proportion
Contextually irrelevant entities. References to genes, environments, or biological processes, which are misidentified as organisms involved in ecological interactions.	Glucan	63.10%
Ambiguity in organism naming, including homonyms and vernacular terms encompassing broad, non‐monophyletic groups.	Algal grazer populations	12.30%
Unresolved names because of qualifiers that could not be safely removed automatically.	Small bullfrog tadpoles	7.86%
Unresolved vernacular names and nouns derived from latin names.	Coleopteran	5.41%
Spelling errors in the source text.	*Chenolibia testudinaria*	5.41%
Taxonomic gaps in the reference database, which remains incomplete for lesser‐known organisms and for some synonyms, and can deliberately exclude non‐accepted taxonomic names used in source texts.	*Neobiscutum*	3.93%
Other, unclear reasons.		1.97%

*Note:* For each reason we provide an example and the estimated proportion in our data using a validation set of 1000 manually checked names.

We excluded outputs from 29 paragraphs in which the model repeatedly generated the same word or phrase until the maximum number of output tokens was reached. Because these cases were rare and generation was capped, they did not measurably affect the overall token consumption or cost. Additionally, we removed 4272 extracted interactions that matched exactly the one‐shot demonstration example included in the prompt template, indicating probable prompt‐example copying (i.e., an example‐leakage artifact) rather than evidence derived from the source paragraph. To merge synonymous nodes and remove potential false positives, we filtered the data to keep only interactions involving taxonomically linked entities. As each interaction has been categorized by the model, we also merged all edges between two nodes belonging to the same category. This resulted in a final interaction network of 35,471 nodes and 144,402 edges (interactions) originating from 112,647 paragraphs, from 36,044 publications.

The global set of extracted interactions were filtered and aggregated at different levels of taxonomic resolution (Table S1). For example, the species level network involved 18,589 different species and 46,467 species‐to‐species interactions distributed in 1295 components (i.e., disconnected subparts of the network). A detailed view of the main component of this network is displayed in Figure 3. Interactions were dominated by parasitism (Figure 3a–c), and the species with the most interactions extracted were species exhibiting a major role in human cultures, such as domestic animals and livestock, or parasites of medical or veterinary interest (Figure 3d).

**FIGURE 3:**
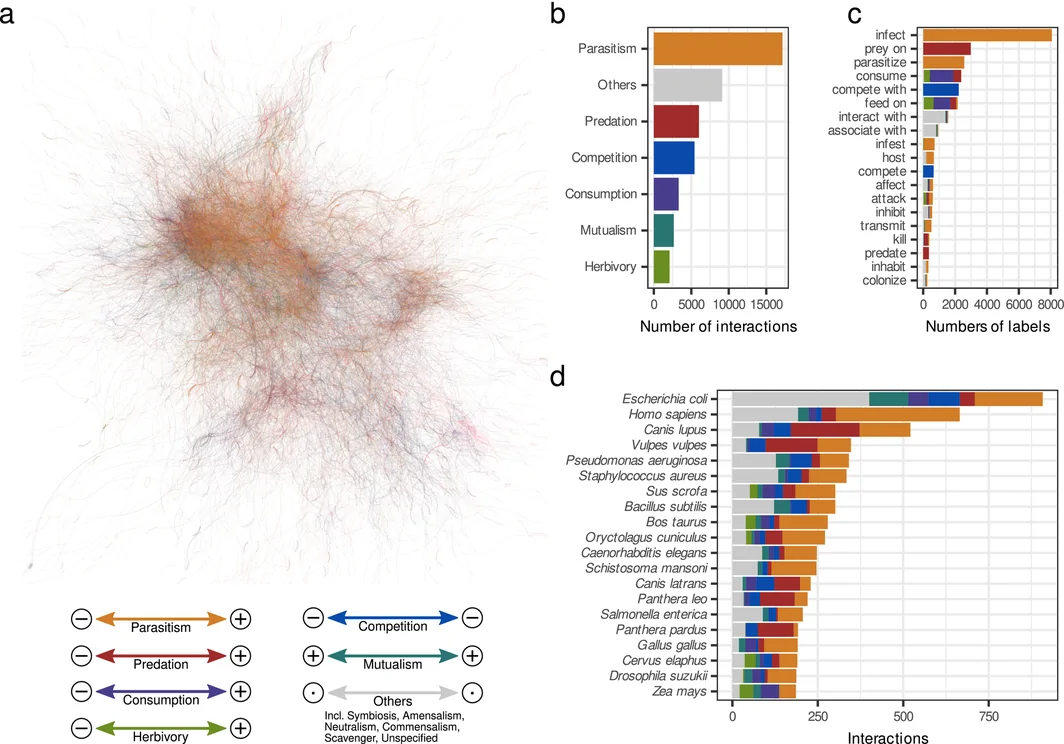
Reconstructed network of interactions at species level. (a) Overview of the main graph component represented using a force‐based (ForceAtlas2) layout. (b) Number of interactions for the different inferred categories across the whole network. (c) Number of extracted labels (raw text extract) and their inferred categories (standardized classes shown in the legend) for the 20 most common labels. (d) Number of interactions and their inferred categories for the 20 most connected species (connectivity computed by counting all interactions in which a species appears, regardless of whether it is source or a target in interactions). In each panel, interaction categories are color‐coded as described in the legend, which also indicates for each category how the partners are affected (positively or negatively).

In the first validation set of 500 randomly selected paragraphs, we manually identified 327 biological interactions. Our automated approach managed to identify 229 of these (true positives), while making 27 errors (false positives). This corresponds to a precision of 89.5% (CI_95%_ [83.8%, 94.2%]), a recall of 70.0% (CI_95%_ [63.1%, 76.8%]), and a F1‐score of 78.6% (CI_95%_ [72.6%, 83.5%]). Of the 500 paragraphs in this randomly sampled validation set, 353 contained no relevant interactions. Among these, not a single instance was found where the workflow falsely detected a species interaction that clearly was not present. While this does not entirely rule out the possibility of hallucination by the LLM, it demonstrates that our prompting and filtering strategy is highly robust against this specific type of error. To make the evaluation more diagnostic, we provide a table of the 98 false negatives (i.e., the interactions present in the manual annotations for which the workflow produced no corresponding record) and the 27 false positives (incorrect extraction) by interaction category (Table S2). We note that per‐category counts are small for several classes, so category‐level statistical interpretations remain difficult. We therefore provide these summaries as descriptive guidance on the dominant issues and the most affected categories. Precision in the stratified validation set was slightly lower (85.0%, CI_95%_ [78.4%, 91.2%]), while recall was higher (88.5%, CI_95%_ [84.7%, 92.1%]) resulting in an F1‐score of 86.7% (CI_95%_ [82.3%, 90.3%]). The performance metrics for each validation set and for the different components of the extraction tasks are shown in Figure 1. An illustrative selection of representative examples of extractions is provided in Table S3.

Across replicate runs, we found a mean agreement of 82% across 125 interactions when retaining the finest available linked taxonomic resolution and a mean agreement of 88% across 22 interactions when restricting to species‐level entities only.

The interactions were described in the source publications with a very rich and more or less specific vocabulary (14,767 unique labels, see Figure 4 for an overview of label‐to‐category associations), demonstrating the importance of a good understanding of the context by the model.

**FIGURE 4:**
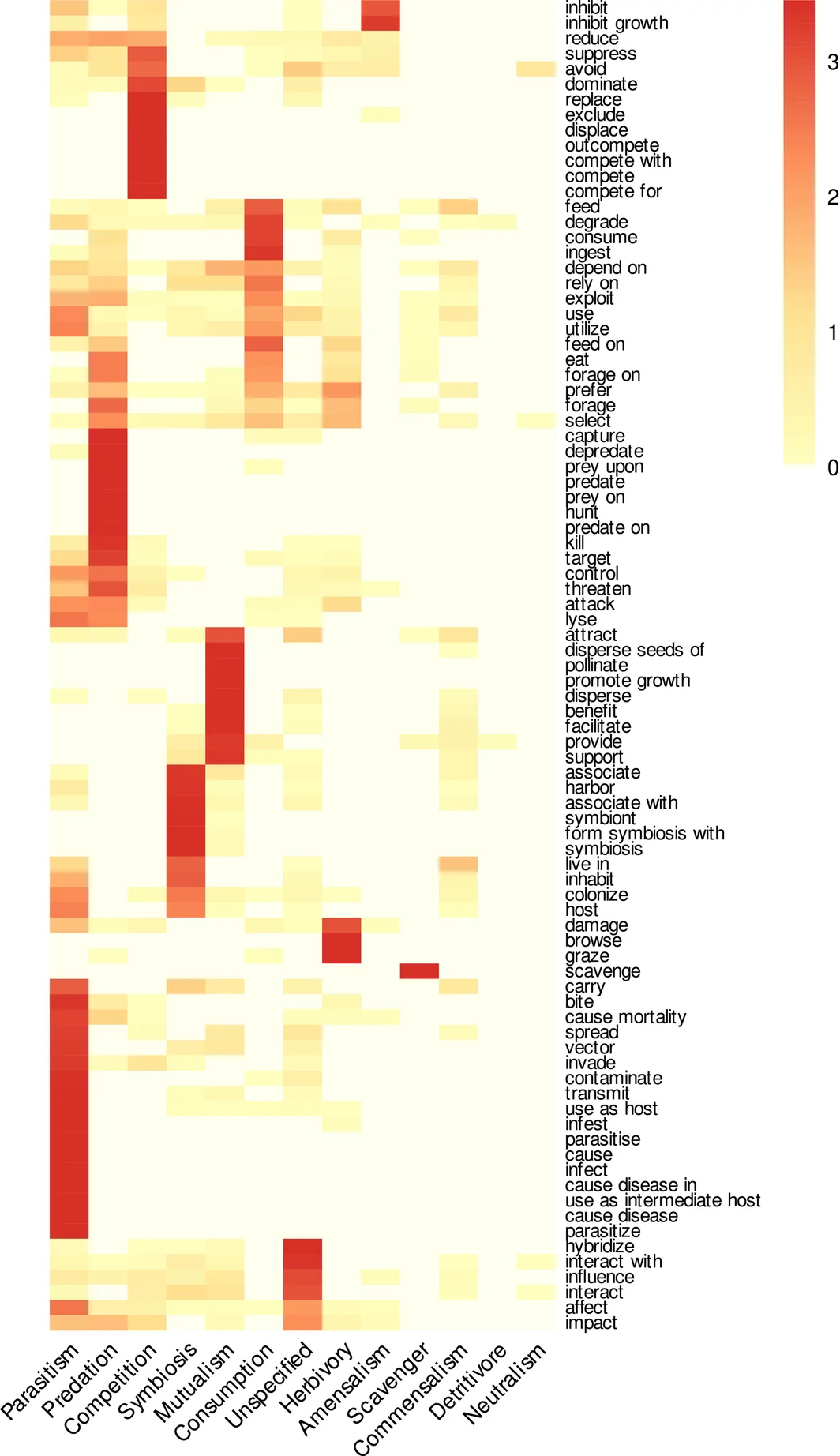
Heatmap showing the association between extracted labels and inferred interaction categories. Values represent the number of times each label has been classified into each category, scaled by row to improve readability. Only the most common labels (extracted more than 250 times) are shown (*n* = 87).

## 4 Discussion

As environmental challenges are multiplying and becoming more pressing, it becomes increasingly important to mobilize all available knowledge in ecology. Here, we demonstrated that this is now within reach and made possible by new developments in artificial intelligence and LLMs. We extracted detailed information on biological interactions, a key parameter in ecology, among thousands of species directly from the scientific literature and across a large full‐text corpus, with the opportunities to further integrate these data into existing products or to produce original ecological research about the structure and properties of this global ecological meta‐network (Delmas et al. 2019; Guimarães 2020) while being clear and transparent about the use of LLMs and the origin of the data. Up to now, access to species interaction data has been one of the most limited and biased, yet one of the most sought‐after data types (Hortal et al. 2015; Poisot et al. 2021). Our contribution lies in the development, implementation, and validation of a robust, scalable workflow that embeds state‐of‐the‐art LLMs to an ecologically complex and heterogeneous corpus and a challenging task. We thus expand beyond recent proof‐of‐concept studies (Castro et al. 2024; Gougherty and Clipp 2024; Scheepens et al. 2024) by demonstrating feasibility at scale, identifying key bottlenecks, and rigorously evaluating model performance across a wide range of interactions and taxa. Our goal is, first and foremost, methodological as we aim to show that structured ecological knowledge can be extracted from the existing literature using current tools, and to lay a foundation for future ecological syntheses built on such automated workflows.

Our study applies the extraction workflow to a large and heterogeneous corpus, which allows us to assess how LLM‐based information extraction behaves in a realistic large‐scale deployment. We estimate extraction performances through manual annotation of two subsets (a random sample and a complementary stratified sample) and use these evaluations to identify the possible bottlenecks and limitations that emerge when scaling the approach. Our method effectively balanced overall correctness with the ability to identify relevant cases, even in challenging scenarios. While the recall (70.0%) suggests room for improvement in detecting all relevant interactions, the relative high precision (89.5%) highlights the robustness of the model in minimizing false positives. Our stratified sample with balanced representation of taxa showed similar performances. Precision with this dataset is minimally lower (85.0%) and could indicate that the best represented and most common taxa are also those best handled by the model. Recall is higher (88.5%), likely because this stratified subset is conditional on the workflow returning at least one interaction and therefore includes no empty output paragraphs. In the non‐stratified evaluation, paragraphs with empty outputs account for most of the missed interactions and therefore reduce recall. Accordingly, the stratified recall should be interpreted as performance given detection, rather than as an unbiased estimate of overall recall. Overall, the analysis of the various components of the extraction process shows that annotation success depends above all on the first step (i.e., the detection of the interaction itself), which is both critical and particularly challenging for the model.

We explicitly instructed the model not to assume prior knowledge about the organisms, but it remains challenging to guarantee that information encoded during pretraining did not influence individual outputs. Such influence may be particularly plausible for common and widely documented interactions, whereas its importance for rare species‐level records remains uncertain (Dorm et al. 2026). Nevertheless, this does not compromise our evaluation of extraction accuracy as an interaction was considered correct only when it was supported by the supplied paragraph, and each extracted record remains traceable to its source text. The model's prior knowledge may have helped it to interpret some paragraphs, but it could not turn an unsupported output into a true positive under our validation procedure. Thus, this uncertainty concerns how the model reached its decision, rather than whether the retained interaction was supported by the text.

Although the extraction relies primarily on an LLM, it is part of a more complex data processing workflow that guarantees the quality of results (Figure 1). First, we implemented a document filtering and preprocessing step, applied before running the LLM, to build the most comprehensive and specific corpus possible. The role of this filter is to target the most relevant documents, hence limiting the number of LLM requests and therefore reducing the financial and environmental costs of the extraction. The technical choices underlying this step are critical, as they directly define the scope and boundaries of the final corpus. While filtering errors do not compromise the quality of the generated data (since irrelevant documents are simply excluded), they may result in the omission of potentially useful source texts. In our case, we adopted a conservative strategy designed to prioritize inclusion, even at the cost of capturing more false positives. Nevertheless, we cannot fully exclude that a small number of valuable documents were mistakenly filtered out. For instance, TaxoNERD, while very effective overall, may have varying performance across taxonomic groups (Le Guillarme and Thuiller 2022). Also, while our keyword list was carefully optimized for exhaustivity, we cannot exclude that it still misses some rare and unconventional interaction formulations. This limitation may be more or less present, depending on the type of corpus analyzed. In less standardized corpora, using different vocabulary or conventions (e.g., abbreviations), it is important to ensure that the keyword filter works and to make the necessary adjustments. Second, we developed a data filtering and postprocessing step, applied after the LLM inference, to improve the quality and harmonize the results by removing false positives and potential hallucinations. This postprocessing combined automated quality check with TEL (to NCBI taxonomic IDs) to filter spurious records and merge synonyms and duplicates. In our experiment, this filtering proved to be necessary to remove the large number of false positives extracted by the model (e.g., interactions involving irrelevant or ambiguous entities).

Importantly, as vast and rich as the scientific literature may be, it is inevitably biased, and the data extracted by our approach precisely reflects these biases. The species most represented in our results include humans ( *Homo sapiens* ), human pathogens (e.g., *Escherischia coli*, *Pseudomonas aeruginosa* , *Staphylococcus aureus* ) and domestic species (e.g., *Canis lupus* , *Sus scrofa* , *Bos taurus* ), all of which have been the focus of particular attention from the scientific community. Data extraction can only highlight biases in the literature and does not correct them. This is an aspect that must always be considered when examining this type of result. Nonetheless, these biases themselves, when systematically analyzed, also offer new opportunities to study patterns in research effort, knowledge gaps and historical data coverage. Thus, in addition to validating our extraction workflow, we illustrate its potential by highlighting a few prominent patterns emerging from the resulting dataset. We also note that our approach can be used to extract other useful information, such as detailed experimental metadata, spatial and temporal contexts, or other qualitative or quantitative measurements, hence greatly expanding the scope of possible applications. Yet we do not aim here to deliver new ecological conclusions, but to enable them by demonstrating that LLMs can extract structured, high‐quality data from the literature at scale and with acceptable precision.

A number of challenges and limitations have also been identified regarding the use of proprietary LLMs for scientific research (Ollion et al. 2024). First, LLMs can pose a problem for reproducibility because outputs can be non‐deterministic, that is, the same query (prompt) might yield different results depending on the model's internal stochastic sampling processes. A potential solution is to perform deterministic sampling by using fixed random seeds. This approach makes outputs more consistent across runs but all conditions (hardware, software, and model parameters) must be controlled, which is difficult to ensure with cloud‐based closed models. To support reproducibility and enable the community to replicate and extend our work, we provide detailed prompts, and all data, code, and filtering procedures. Moreover, our agreement analysis indicates that, for this extraction task, this corpus, and this model, the workflow outputs are stable for most interactions. Importantly, repeatability can also provide a pragmatic confidence proxy as interactions that appear consistently across many replicates can be treated with higher‐confidence. This idea can naturally be extended to cross‐model consensus, where agreement across different LLMs could further mitigate specific model weaknesses. Secondly, proprietary models are essentially black boxes. The data and methodology used to train them are generally kept confidential by the developing companies. Such opacity means researchers cannot directly audit potential biases introduced by these design choices. Analyzing the model's outputs carefully and performing a thorough validation, as done in the present study, is therefore indispensable for any scientific application of this type of approach. Validation does not, however, resolve the constraints of proprietary models.

The above challenges are solvable, at least in part, through the use of small open models that can be optimized and used locally. Small‐scale LLMs—when appropriately fine‐tuned—can achieve impressive results across different applications (Alizadeh et al. 2024; Carammia et al. 2024; Zhang et al. 2024). The advantages of this approach are many (Chen and Varoquaux 2024): greater independence, greater transparency, better control on reproducibility, and potentially lower costs and environmental imprint. Moreover, running a locally hosted model enables the processing of documents covered by more restrictive licenses (Sommers et al. 2024). However, selection, deployment, optimization and fine‐tuning of such models may require a certain amount of expertise and resources, although the development of cloud‐based solutions is lowering these technical barriers. Despite these new challenges, it is clear that the impact of LLMs will be all the greater if they are put directly into the hands of scientists and make their way into laboratories (Chen and Varoquaux 2024; Ollion et al. 2023). In this respect, small‐scale fine‐tuned LLM represents a promising direction.

Our approach can augment the way we interact with scientific literature, fitting in with the epistemological revolution initiated by the emergence of LLMs (Morera 2024). Through our results we show that a new level of qualitative and quantitative synthesis of the available knowledge can be achieved. Building on recently published proof‐of‐concepts (Castro et al. 2024; Gougherty and Clipp 2024; Scheepens et al. 2024), our study grounds the use of LLMs for synthesis sciences and by demonstrating that we can extract accurate structured information from scientific publications on a large scale, it opens the way to a broad range of applications. Whether for building databases or conducting meta‐analyses, the number of questions that can be addressed in ecology is significant. A straightforward and promising application is the extraction of multiple layers of ecological information, such as habitat context, experimental conditions, or spatial and temporal metadata, that are often embedded in unstructured narrative form, and which could be turned into more comprehensive and searchable databases. However, the generation of comprehensive and scientifically accurate databases should not be interpreted as the automatic result of a simple query to an LLM. It requires explicit quality controls, uncertainty handling, and validation on human‐annotated samples. It is also important to note that the present experiment represents a snapshot of a rapidly evolving technology, as the extraction was performed using a model released in 2024. Since then, substantial advances (e.g., reasoning capabilities, agentic workflows) have created new opportunities to further improve extraction performances. Where high confidence is required, a possible option is to adopt consensus strategies (e.g., multiple extraction passes and retaining only records that are stable across runs, and/or agreement across different models). As an additional promising direction, one can use an independently prompted LLM to review extracted records against the source text (Parkinson et al. 2026). Such model‐based review may help flag unsupported or ambiguous records, although correlated errors between models mean that it should complement, rather than replace, validation against human annotations. It would also be informative to replace or complement the LLM extraction step with a rule‐based algorithm and benchmark when, and at what engineering cost, such heuristics can match or fall short of LLM performance. Simple rule‐based approaches would likely not provide a meaningful comparison because they cannot resolve taxon pairing, directionality, negation, or passive voice, whereas developing a realistic rule‐based system capable of handling these features would require substantial corpus‐specific engineering that is much more costly to develop, maintain, and port across domains and questions than an LLM‐based approach. Automated data extraction also paves the way for living syntheses, that is, studies that are continually updated and whose results and conclusions evolve as new primary results are made available (Berger‐Tal et al. 2024). In the context of the ongoing biodiversity crisis where scientists are confronted with an inflation in the number of publications and the dispersal of information, advancing ecological data access and extraction to a new level will help to better understand and model current and future species distribution and interactions and guide critical management decisions.

## Author Contributions

**François Keck:** conceptualization (lead), data curation (lead), formal analysis (lead), methodology (lead), software (lead), visualization (lead), writing – original draft (lead). **Henry Broadbent:** writing – review and editing (equal). **Florian Altermatt:** funding acquisition (lead), project administration (lead), supervision (lead), writing – review and editing (equal).

## Funding

This work was supported by the Universität Zürich and the Schweizerischer Nationalfonds zur Förderung der Wissenschaftlichen Forschung (grant 310030_197410).

## Conflicts of Interest

The authors declare no conflicts of interest.

## Supporting information


**Appendix S1:** ece374626‐sup‐0001‐Supinfo1.docx.

## Data Availability

The publications used to produce the results are all part of the PMC Open Access Subset collection (https://www.ncbi.nlm.nih.gov/pmc/tools/openftlist/). All data extracted and all code used for analysis are openly available on GitHub (https://github.com/fkeck/gpt_interactions) and archived on Zenodo (https://doi.org/10.5281/zenodo.21397883).
