# Supplementary material for: A Scalable Workflow for Extracting Species Interactions From Ecological Literature Using Large Language Models
Source: Ecol Evol. 2026 Oct 27;16(11):e74626. doi: 10.1002/ece3.74626 (PMC13713224; doi:10.1002/ece3.74626)
Supplement: Supplementary file 1 [file ECE3-16-e74626-s001.docx]

**Supporting Information for:**

A scalable workflow for extracting species interactions from ecological literature using large language models

François Keck^1,2^, Henry Broadbent^1,2^, Florian Altermatt^1,2^

^1^ Department of Evolutionary Biology and Environmental Studies, University of Zurich, Winterthurerstr. 190, CH-8057 Zürich, Switzerland

^2^ Eawag, Swiss Federal Institute of Aquatic Science and Technology, Department of Aquatic Ecology, Überlandstrasse 133, CH-8600 Dübendorf, Switzerland

# Methods S1

Below we provide a summary of the LLM-based workflow technical details. The exact implementation is provided in the versioned code repository (see Data and Code Availability Statement).

### **1. Model and API parameters**

- Model: OpenAI GPT-4o
- Model version: gpt-4o-2024-05-13
- Model parameters:
  - temperature: 0
  - n: 1
  - top_p: 1
  - max_tokens: 4,096
  - seed: null
  - stop: null
  - frequency_penalty: 0
  - presence_penalty: 0
  - response_format: raw text
  - logprobs: false
  - top_logprobs: null

### **2. Prompt**

**SYSTEM**

Use the following step-by-step instructions to respond to user input.

Step 1: Identify all biological organisms from their vernacular or taxonomic names.

Step 2: For each pair of organisms found in Step 1, extract (if they exist!) their pairwise biotic interactions. Typical biotic interactions are: competition, predation, consumption, mutualism, parasitism, symbiosis. Do NOT assume any prior knowledge about the organisms.

Step 3: Classify each extracted interaction into one of the following categories: ['competition', 'amensalism', 'neutralism', 'commensalism', 'mutualism', 'parasitism', 'predation', 'herbivory', 'symbiosis', 'scavenger', 'detritivore', 'unspecified', 'consumption']. Choose only one category for each interaction and strictly adhere to the provided list.

Extracted data MUST follow the structured schema below:

```TypeScript

interaction: Array<{ // Biotic interaction between two organisms found in Step 1

from: string // The name of the organism that perform the interaction

to: string // The name of the organism that undergoes the interaction

label: string // Active verb describing the biotic interaction performed by 'from' on 'to'

category: string // The category of interaction as inferred in Step 3

}>

```

The 'from' and 'to' values MUST only contain names of organism found in Step 1. Interactions are directed, so the order of the 'from' and 'to' values IS IMPORTANT.

Please output the extracted data in CSV format in Excel dialect and use a | as delimiter. Do NOT add clarifications. Do NOT add columns that do not appear in the schema above. If there is no biotic interaction in the user input, output an empty CSV.

**USER**

Rodents include lemmings and beavers. Hawks hunts lemmings. Bats are frugivores on Carica papaya. Festuca is commonly grazed by Capra ibex.

**AGENT**

from|to|label|category

hawks|lemmings|hunt|predation

bats|Carica papaya|feed on|consumption

capra ibex|festuca|graze|herbivory

**USER**

<Paragraph content>

### **4. Parsing rules**

Individual responses were processed as follows:

- If the header from|to|label|category is not present in the first line, remove the first line.
- Remove final `\n````.
- Convert outputs consisting only of ``` into an empty string.
- Drop empty contents.
- If the remaining content still lacks the exact header, replace the entire response with a header-only table.
- Parse using R read.delim(text=..., sep="|"), with the first line as column names.
- Convert inferred non-character columns to character.
- On any parsing exception, substitute a header-only zero-row table.
- Bind parsed rows.

### **5. Retry and error handling**

For the OpenAI extraction itself, no specific retry and error handling was needed (all queries were processed successfully on the server). Parsing failures become empty tables without logging.

Retries were implemented for downstream taxonomic linking:

- Taxon resolution: three total attempts, 15-second timeout per attempt; ordinary errors wait approximately six seconds before retrying; exhaustion returns NA. See function resolve_taxo_retry() defined in FUN_taxonomic_entity_linking.R.
- NCBI classification: approximately 100 UIDs per chunk, three total attempts, 30-second timeout; ordinary errors wait approximately eleven seconds.
- Taxonomic resolution checkpoints are saved every 500 names.

### **6. Postprocessing filters**

The following filters were applied to the final dataset:

- Remove the three example interactions from the one-shot learning.
- Normalize Unicode dashes, apostrophes and quotation marks.
- Remove vernacular qualifiers such as “species”, “strains”, “larvae”, “hosts”, “parasites”, habitat/diet qualifiers and OTU/MOTU.
- Remove numbers, parenthetical content, uncertainty markers such as cf., aff., sp., spp., and trailing punctuation from taxon names.
- Map phage-like names to unclassified bacterial viruses.
- Drop cleaned taxon names of at most two characters.
- Retain only one original name for each identical cleaned name.
- Resolve names through ITIS/EOL, Global Names, NCBI and Wikipedia-based fallbacks.
- Drop unresolved UIDs and retain only one record per UID.
- Keep taxonomy ranks: superkingdom, phylum, class, order, family, genus, species.
- Retain an interaction only when both endpoints resolve, and only at ranks available for both endpoints.

\b[Pp]redat[a-z]*\b

\b[Hh]unt[a-z]*\b

\b[Aa]ttack[a-z]*\b

\b[Kk]ill[a-z]*\b

\b[Cc]onsum[a-z]*\b

\b[Ff]orage[a-z]*\b

\b[Gg]raz[a-z]*\b

\b[Ee]xploit[a-z]*\b

\b[Ee]at[a-z]*\b

\bate\b\

\b[Ff]eed[a-z]*\b

\b[Ff]ed\b

\b[Ff]ood\b

\b[a-z]*troph[a-z]*\b

\b[Pp]rey[a-z]*\b

\b[Pp]ollinat[a-z]*\b

\b[Dd]ispers[a-z]*\b

\b[Ss]pread[a-z]*\b

\b[Pp]arasit[a-z]*\b

\b[Ii]nfect[a-z]*\b

\b[Ii]nfest[a-z]*\b

\b[Cc]oloni[sz][a-z]*\b

\b[Tt]ransmit[a-z]*\b

\b[Vv]ector[a-z]*\b

\b[Hh]ost[a-z]*\b

\b[Pp]athogen[a-z]*\b

\b[Rr]esist[a-z]*\b

\b[Cc]ommensal[a-z]*\b

\b[Ss]ymbio[a-z]*\b

\b[Mm]utualis[a-z]*\b

\b[Cc]ommensalis[a-z]*\b

\b[Aa]mensalis[a-z]*\b

\b[Cc]ompet[a-z]*\b

\b[Oo]utcompet[a-z]*\b

\b[Rr]ecycl[a-z]*\b

\b[Dd]ecompos[a-z]*\b

\b[Dd]egrad[a-z]*\b

\b[Nn]utrient[a-z]*\b

\b[Rr]esource[a-z]*\b

\b[Pp]romote\b

\b[Ii]nhibit\b

\b[Ii]nteract[a-z]*\b

\b[Pp]artner[a-z]*\b

**Data S1.** List of keywords used in the pre-filtering to select the relevant paragraphs. The keywords are presented as regular expression strings allowing extended matching. The part of the expression [Xx] allows words to start with a capital or lowercase character. The part of the expression [a-z]* allows for zero or more additional lowercase letters, capturing any word that extends the base keyword with common suffixes like "or", "ion", or "ing". The use of the \b flag at both the beginning and end ensures that the match only occurs at full word boundaries, meaning it will not match partial words embedded within longer strings.

| **Taxonomic level** | **Nodes** | **Edges** | **Components** |
| --- | --- | --- | --- |
| Mixed (all) | 36,471 | 144,402 | 933 |
| Superkingdom | 4 | 157 | 1 |
| Phylum | 127 | 5,201 | 2 |
| Class | 353 | 9,772 | 2 |
| Order | 1,140 | 26,345 | 11 |
| Family | 4,118 | 42,744 | 58 |
| Genus | 12,980 | 56,404 | 390 |
| Species | 18,589 | 46,467 | 1,295 |

**Table S1.** Summary statistics of the extracted network aggregated at different taxonomic levels. The nodes correspond to individual taxa, the edges represent the interactions between the taxa, and the components are the subparts of the main graph that are not connected together.

| **Category** | **Correct** | **Missed** | **Incorrect** |
| --- | --- | --- | --- |
| Amensalism | 8 | 2 | 1 |
| Commensalism | 1 | 1 | 0 |
| Competition | 7 | 1 | 0 |
| Consumption | 18 | 11 | 2 |
| Detritivore | 2 | 0 | 0 |
| Herbivory | 11 | 3 | 1 |
| Hybridization | 1 | 0 | 0 |
| Mutualism | 12 | 0 | 0 |
| Parasitism | 111 | 64 | 1 |
| Predation | 39 | 15 | 0 |
| Symbiosis | 10 | 1 | 0 |
| Unspecified | 9 | 0 | 1 |

**Table S2.** Missed interactions and errors by interaction category. For each interaction category, the table reports the number of correctly extracted interactions, the number of missed (non-detected) interactions and the number of erroneously extracted interactions, defined as extracted records that disagree with the manual annotation in any component. Counts are reported as absolute numbers based on the manually evaluated non-stratified set of paragraphs.

| **Source text** | **LLM Extraction** | **Comment** |
| --- | --- | --- |
| [Kadyrov et al. 2016. Zookeys] This species occurs in Kyrgyzstan, Tajikistan, and Uzbekistan and is endemic to the region of western Tian Shan Range. Ropalopusnadari is a polyphagous species, which usually inhabits growing wild fruit trees in the upper zone of deciduous forests (Fig. 4D). This species most frequently inhabits trunks and boughs of the wild apple tree Malussieversii. Its larval development usually takes two years. Adults are found from June to July. After they emerge, the imagines are unwilling to fly and generally stay on the host plant and only visit flowers of Ferula spp. and Prangos spp. occasionally. | NULL | Empty output (Missed interactions) |
| [Amorim et al. 2018. Ecology and Evolution]  The long‐term persistence of a species in a given landscape is conditional on the availability of resources at the appropriate temporal and spatial scales. As the resources and their spatial distribution change over time, it is highly likely that species’ distributions change accordingly to track such resources. For instance, the food resources required by a species often vary along the life cycle and among life stages, often associated to spatial changes in food availability. Such changes may be overcome either by species following the resources through different habitats, or by different habitats becoming available at the optimal time for a given species. Both strategies will lead to temporal variation in species diversity and abundance across the landscape, which should be particularly evident for highly vagile organisms such as bats and birds. Understanding such spatiotemporal patterns is paramount for conservation, due to the need of protecting all habitats providing the resources to fulfill species’ requirements across the whole year. However, such information is seldom available as most studies only provide snapshots from a single season or pool yearly data together into a single analysis, generally disregarding seasonal variations. | NULL | Empty output  (No missed interactions) |
| [Prkno et al. 2017. Viruses]  By having a look on the course of infection through the alpaca herds, in all four herds, infection began to appear in one single flock and distributed later among other flocks in two herds. Herd III exhibited more exemplary infection courses and possible ways of transmission. Likely, virus entered the herd by initially infecting one individual through water or fodder contaminated by infectious rodent feces or urine. This theory was nicely affirmed by the CPXV-infected common vole in the water bucket of flock 3. Serology data reveal that after one animal got infected in flock 3, sequentially, all other animals of this flock had seroconverted. The virus was meanwhile also transmitted to the mare flock and one by one the animals seroconverted. As alpacas are so called “distance animals”, avoiding close contact to each other and not performing pair grooming, direct animal contact as way of virus transmission does not seem obvious (only in case of biting each other, as stallions do while fighting, or in the case of spitting on each other). The fact that none of the stallions of flock 2, which could have direct animal contact to the stallions of flock 3 through the fence, showed positive serology results, supports this. Typical comfort behaviors of SACs are scraping themselves against items in the direct environment (brooms, wood, etc.) and wallowing in the sand. In addition, animals of one flock use the same water bucket and fodder racks; there was a CPXV DNA-positive tested broom in the stable of flock 1, so virus transmission through indirect contact appears to be very likely. As infectious crusts of pox lesions have a high tenacity in the environment, indirect transmission, whether by alpaca-associated utensils, by fodder (hay and straw), or maybe also through contact to humans might be possible for a certain time. While these scenarios fit well to the situations in herds I and II, there is no reasonable answer why only one alpaca mare in herd IV got infected and how it became infected. | rodents -> alpacas [infect/parasitism]  common vole -> alpacas [infect/parasitism] | Incorrect interpretation (Wrong interactions) |
| [Bouguerche et al. 2021. Parasite]  Similarly, a few other diclidophorids were described from parasitic crustaceans: Allodiclidophora squillarum [syn. Mesocotyle squillarum Parona & Perugia, 1889] on the bopyrid Bopyrus squillarum Latreille, 1802 off Italy; Allodiclidophora charcoti [syn. Cyclobothrium charcoti Dollfus, 1922] on a female Ceratothoa oestroides from the buccal cavity of Trachurus trachurus and of Boops boops, caught from Spain and Monaco, respectively; and Diclidophora merlangi Krøyer, 1838 [syn. Dactylocotyle merlangi Kuhn, 1829] collected on Ceratothoa oestroides from the buccal cavity of Boops boops from Italy. | Diclidophora merlangi -> Ceratothoa oestroides [parasitize/parasitism] | Correct but incomplete extraction (Missed interactions) |
| [da Silva & Hernández. 2015. PlosOne]  Another important issue associated with the high movement rates found for large-diurnal tunneler species is the predation pressure that one may expect to be more important at certain times of day, depending on the diel activity of predatory species. For nocturnal species, short periods of flight may be expected to reduce the pressure of visual predators. The opposite is expected for diurnal colorful species, such as Coprophanaeus saphirinus, which travels greater distances by avoidance of visual predators due to body coloration that camouflages or advertises their toxicity to predators. Among the predators we can generally cite some birds (e.g. owls), mammals (e.g. bats), spiders and species of beetles from the families Carabidae and Staphylinidae. However, predation on dung beetles still needs to be further investigated, because its effect on species behavior or community structure may be minimal or insignificant, as suggested by the meager available information. | owls -> Coprophanaeus saphirinus [prey on/predation]  bats-> Coprophanaeus saphirinus[prey on/predation]  spiders -> Coprophanaeus saphirinus [prey on/predation]  Carabidae -> Coprophanaeus saphirinus [prey on/predation]  Staphylinidae -> Coprophanaeus saphirinus [prey on/predation] | Correct extraction |

**Table S3.** Examples of LLM-based interaction extraction. Each row reports the source text excerpt, the corresponding structured extraction returned by the LLM (before TEL), and a brief comment about the quality of the result.

**Figure S1.** Performance metrics estimated using a) the random dataset and b) the stratified dataset. For each set, the first plot shows the performances for the complete annotation of the interactions. Subsequently, the metrics are detailed for their individual components: the detection of the interactions (pairs of species), the label of the interactions, and the classification of the interactions. Error bars indicate 95% confidence intervals.
